# Modified CAMBRA Protocol for Caries Risk Assessment in Children Aged 6 to 14 Years

**DOI:** 10.3390/dj13110530

**Published:** 2025-11-12

**Authors:** Esther Pérez de Mora, Ángel Luis Formoso-Veloso, Marcela Arenas-González, Asunción Mendoza-Mendoza, David Ribas-Pérez

**Affiliations:** 1 Department of Pediatric Dentistry, Faculty of Dentistry, University of Seville, 41009 Seville, Spain; estperde@us.es (E.P.d.M.); amendoza@us.es (A.M.-M.); dribas@us.es (D.R.-P.); 2 Department of Orthodontics, Faculty of Dentistry, University of Seville, 41009 Seville, Spain; spain.aformoso@us.es

**Keywords:** CAMBRA, dental caries, hygiene, diet, risk of dental caries

## Abstract

**Background/Objectives**: This study aimed to propose a modified CAMBRA protocol for caries risk analysis in a pediatric population, adapted to their needs and habits. **Methods**: A descriptive and analytical observational study was conducted after obtaining approval from the Ethics Committee. Caries risk was determined using the CAMBRA Questionnaire and the modified University of Seville Questionnaire (CAMBRA-OP Questionnaire). **Results**: The final study sample consisted of 300 patients aged 6 to 14 years, of whom 54% were boys and 46% were girls. The distribution of caries risk according to CAMBRA was as follows: 33% low, 7% moderate, 48.6% high, and 11.3% extreme. The distribution of caries risk according to the CAMBRA-OP was as follows: 27.7% low, 12.3% moderate, 48.7% high, and 11.3% extreme. Correlating both tests, a statistically significant association was observed between the caries risk determined by the CAMBRA and CAMBRA-OP, observing a strong correlation between both systems (*p* = 0.001; Cramer’s V = 0.826). The two diagnostic models exhibited high concordance (0.815 [*p* = 0.001]) using Cohen’s Kappa index. **Conclusions**: This tool is focused on this age group and is easily interpretable by professionals, thus contributing to more effective and personalized prevention of dental caries in childhood.

## 1. Introduction

Caries is now understood as a biofilm-related disease caused by an imbalance in the oral microbiome, leading to the dominance of acid-producing bacteria in response to frequent sugar exposure. It is recognized as a dynamic, chronic, non-contagious process that results in the progressive demineralization of tooth hard tissues [1,2,3,4,5,6].

According to the World Health Organization’s Global Oral Health Status Report (WHO, 2022), dental caries remains one of the most prevalent noncommunicable diseases worldwide, affecting both children and adults [7].

Since the mid-1990s onwards, more structured and evidence-based systems for caries risk assessment began to emerge. One of the most influential was the Cariogram, developed by Bratthall (1996) [8] in Sweden. This computer-based program enabled a graphical representation of the probability of avoiding caries, based on multiple biological, behavioral and protective factors. It served as a visual and interactive analytical tool that facilitated communication with patients and supported clinical decision making [9,10].

In a similar vein, the American Dental Association (ADA) [11] published, towards the end of the 1990s, one of the first institutional caries risk assessment guidelines in the United States of America: the ADA Caries Risk Assessment Form, considered a direct precursor to the CAMBRA (Caries Management by Risk Assessment) system [11,12].

The development of the Caries Risk Semaphore is also noteworthy. Introduced in the 2000s in Europe, particularly in Spain and Latin America, it became a practical clinical tool based on a color-coded system (green, yellow, and red) that classified patients as low, moderate, or high risk. This system allowed for immediate visual interpretation and supported an individualized preventive strategy [13,14].

Likewise, the American Academy of Paediatric Dentistry (AAPD) [15] developed specific caries risk assessment forms for pediatric populations, with different versions for children aged 0–5 years and those aged six years and older. These forms, which are periodically updated, incorporate biological, behavior, and environmental factors, and currently serve as an international reference tool in pediatric dentistry [14].

Complementarily, the European Academy of Paediatric Dentistry [16] has published updated recommendations on the assessment and management of caries risk in the European pediatric population, emphasizing the importance of individualized preventive approaches.

Collectively, these models marked the transition towards preventive, evidence-based dentistry focused on individual risk stratification, paving the way for the development of more comprehensive and scientifically grounded systems such as CAMBRA.

The CAMBRA (Caries Management By Risk Assessment) Questionnaire, developed by the California Dental Association in 2002 [15], is a tool that assesses the risk of caries based on patient age and dental plaque presence and type. The original model establishes four risk categories: low, moderate, high, and extremely high. This approach includes a clinical evaluation of caries lesions and proposes specific interventions for each risk level [9,10,13,15]. This protocol facilitates the classification of patients according to their risk of caries, allowing for the implementation of personalized treatments based on protective factors. This preventive and interceptive protocol covers the entire pediatric population, including those considered at low risk, who may also benefit from the protocol preventive measures [9,10,16,17]. The original CAMBRA Questionnaire considers patients older than 6 years of age, where the mixed dentition begins with the eruption of the first permanent molars and incisor replacement. Its use is generalized for all patients in this pediatric age group, since they also have greater neurological maturity [17].

Therefore, we consider that the CAMBRA Questionnaire has certain limitations when applied to children. In our experience, this instrument omits valuable information related to dietary and oral hygiene habits, which we consider essential for proper risk assessment. We also believe that complementary procedures, such as *Lactobacilli* and *Streptococcus* cultures, may be impractical in daily practice, failing to provide critical information that justifies their systematic use in routine clinical settings [18,19,20,21,22,23].

Therefore, we believe that it is necessary to develop more specific caries risk assessment tools that are adapted to the characteristics, habits, and needs of different population groups, such as children.

This study aimed to assess the concordance between a pediatric adaptation of the CAMBRA questionnaire and the original protocol in children aged 6–14 years.

## 2. Materials and Methods

### 2.1. Study Type and Study Environment

This cross-sectional, observational, and analytical study was approved by the Research Ethics Committee of the University of Seville. The sample consisted of 300 children residing in the province of Seville who had no known allergies to any product used in the study. The sample size was calculated with a 95% confidence interval (α) and adequate statistical power (β), in accordance with methodological standards [24]. Patients undergoing orthodontic treatment, those with systemic pathologies, or on daily medication were excluded from the study. Informed consent from the legal guardians authorizing the use of personal and clinical data for scientific purposes and assent from the child were obtained.

Caries risk was assessed in a pediatric population by comparing two protocols: the original CAMBRA Questionnaire and a modified questionnaire developed by the research team at the University of Seville (CAMBRA-OP Questionnaire). The clinical, biochemical, and behavioral variables were collected and analyzed.

### 2.2. CAMBRA Questionnaire

The original CAMBRA questionnaire was initially used to determine the caries risk in the study sample. This protocol classifies risk into four levels (low, moderate, high, and extremely high) and includes the collection of salivary samples to determine buffer capacity and salivary flow.

### 2.3. CAMBRA-OP Questionnaire

The CAMBRA Questionnaire was modified to develop a more specific tool adapted for the pediatric population, resulting in the CAMBRA-OP Questionnaire (University of Seville), aimed at children aged 6 to 14 years (Appendix A). This study included salivary buffer capacity and salivary flow tests (Saliva-Check Buffer, GC International AG, Luzern, Switzerland), which were part of the original CAMBRA Questionnaire. Relevant variables, such as diet quality and the modified Quigley-Hein plaque index, were incorporated, whereas *Lactobacillus* and *Streptococcus* cultures were excluded due to their cost and impracticality [25,26].

Certain items from the original CAMBRA Questionnaire were removed as they are unsuitable for the pediatric population, such as the presence of exposed roots, drug use, or uncommon treatments for children, including chlorhexidine varnish, professional fluoride, arginine-based toothpaste, or habitual xylitol consumption. These measures are not part of routine preventive care recommended for the general population of children and are infrequently applied by both healthcare professionals and caregivers.

The inclusion of specific objective elements, such as the modified Quigley-Hein plaque index, salivary pH, social environment, parental supervision, and dietary habits in the CAMBRA-OP Questionnaire, was based on robust empirical evidence [22,23,24,25,26,27] linking these factors to caries risk in the pediatric population. Plaque accumulation and salivary characteristics are well-established biological indicators of caries susceptibility [22,25,26]. Likewise, parental supervision and family environment significantly influence children’s oral hygiene practices and dietary behavior [25,26]. The selection of these elements aimed to address limitations in the original CAMBRA protocol, which lacked sensitivity to behavior and environmental determinants relevant to children under 14 years. These additions enhance the clinical relevance, predictive capacity, and applicability of the questionnaire across diverse pediatric settings.

The CAMBRA Questionnaire, including the CAMBRA-OP version, assesses caries risk by assigning scores to risk and protective factors, which increase or decrease the total score, respectively. The cumulative score classifies patients as low, moderate, high, or extreme risk, providing guidance for preventive measures and an individualized management plan.

The development of the CAMBRA-OP Questionnaire is justified by the need for a pediatric specific assessment tool that goes beyond purely clinical indicators. By incorporating measurable dietary, behavior, and familial factors, and prioritizing measures feasible for routine pediatric practice, this instrument aims to provide a realistic and context-sensitive evaluation of caries risk, enabling the early identification of high-risk children and guiding individualized preventive interventions. This approach addresses the limitations of previous tools, allowing clinicians to design strategies that are both practical and individualized to the realities of the pediatric population.

### 2.4. Cross-Sectional Concordance Analysis

The collected data were initially recorded in a Microsoft Excel spreadsheet, including identification codes, personal information, and values for the variables under analysis. To ensure confidentiality, each participant was assigned a unique numerical code, with only the principal investigator having access to the correspondence linking codes to personal data. The dataset was subsequently exported to IBM SPSS Statistics v.29, where it underwent thorough validation to ensure consistency and integrity, with incomplete or erroneous records being excluded.

The statistical analysis was performed in three main stages. First, descriptive statistics were used to summarize and characterize the distribution of the study variables across the different groups. Second, the strength of association between categorical variables was evaluated using Cramer’s V coefficient, which ranges from 0 (no association) to 1 (perfect association) and is interpreted as negligible (0.00–0.10), weak (0.11–0.20), moderate (0.21–0.40), strong (0.41–0.60), very strong (0.61–0.80), and almost perfect (0.81–1.00). Interexaminer agreement was assessed using Cohen’s Kappa (κ) coefficient (almost perfect (0.81–1.00) agreement).

The internal consistency of the measurement instruments was evaluated using Cronbach’s alpha coefficient. Additionally, the chi-square (χ^2^) test was applied to evaluate the statistical relationship between categorical variables. Finally, regression analysis was conducted to identify factors associated with the prevalence of dental caries.

For all analyses, a 5% significance level (α = 0.05) and 20% beta error (β = 0.20) were used, resulting in 80% statistical power to detect true differences in dental caries prevalence.

## 3. Results

**Figure 1:**
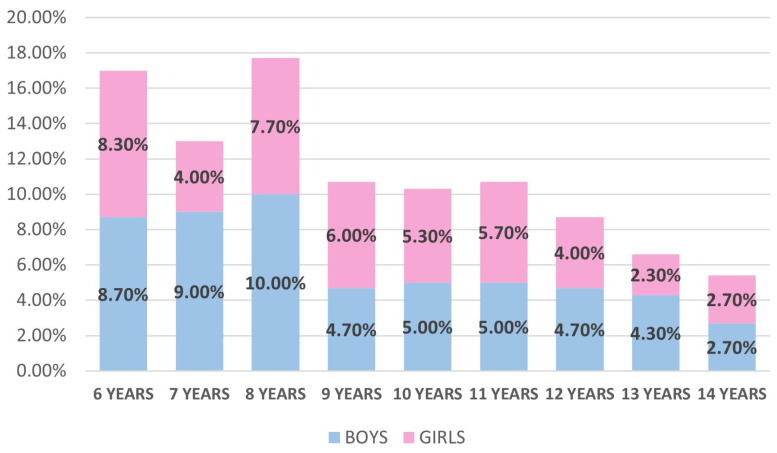
Diagram of sample distribution by age and gender.

Within the sample, 161 (54%) and 139 (46%) patients were male and female, respectively. The age distribution was fairly even between the ages of 6 and 14 years, with slight variations between genders. In particular, the youngest age groups (6 to 8 years) had a higher proportion of boys than girls, whereas in some middle-age groups (9 to 11 years), the proportion of girls was slightly higher or similar to that of boys. The numbers tended to equalize again in the oldest age groups (12–14 years), with small gender differences (Figure 1).

The first table presents information on caries risk as determined by the CAMBRA Questionnaire and the CAMBRA-OP Questionnaire. The results are broken down by gender within the selected sample (Table 1).

The results show that a considerable proportion of the assessed child population is at high risk for dental caries according to both the CAMBRA Questionnaire (48.7%) and the CAMBRA-OP Questionnaire (48.7%). A smaller proportion presented low risk (33% and 27.7%, respectively), while moderate and extreme risk categories were less frequent. The distribution by gender was similar, with boys and girls showing comparable proportions across risk levels (Table 1).

In boys, the low risk percentage decreased from 32.1% with the CAMBRA Questionnaire to 13% with the CAMBRA-OP Questionnaire, whereas the moderate risk percentage increased from 6.8% to 8%. High and extreme risks remained constant, but the percentage changed significantly: high risk increased from 50% to 27.3%, and extreme risk increased from 10.5% to 5.7%. In girls, low risk decreased slightly (from 34.1% to 14.7%), as did moderate risk (7.2% to 4.3%). High risk showed a decrease in percentage, from 12.3% to 21.3%, and extreme risk remained the same in both questionnaires (5.7%) (Table 1).

**Figure 2:**
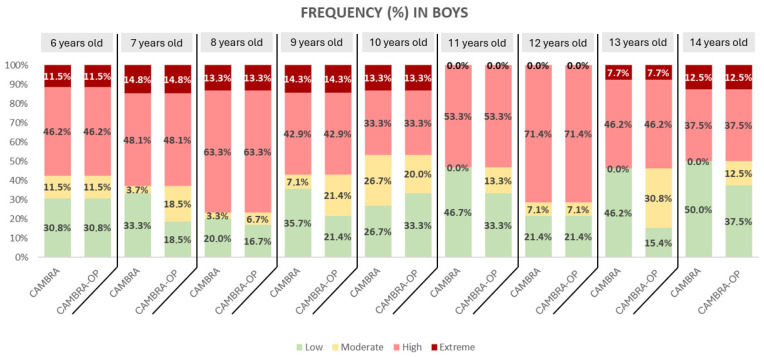
Frequency of the CAMBRA Questionnaire and CAMBRA-OP Questionnaire in boys.

In Figure 2, boys show a predominantly high caries risk (light red) between the ages of 8 and 12, reaching over 60% at some ages. Extreme risk (dark red) is visible between the ages of 6 and 10 years, but almost completely disappears after age 11. In contrast, low and moderate risk (green and yellow, respectively) are more prevalent from ages 13 and 14 onwards, indicating a trend of improvement toward adolescence.

**Figure 3:**
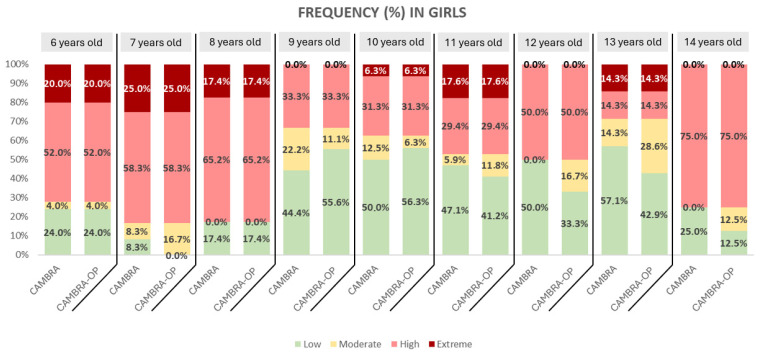
Frequency of the CAMBRA Questionnaire and CAMBRA-OP Questionnaire in girls.

In girls, the pattern is similar, although with a more marked extreme risk at early ages (6 to 8 years) and a predominance of high risk at almost all ages. Unlike boys, girls maintain more consistent high risk percentages until age 14, with less representation of low risk compared to boys (Figure 3).

Cohen’s Kappa index showed high concordance between both diagnostic models, with a Kappa value of 0.815 (*p* = 0.001), which reinforces their reliability, consistency, and reproducibility in clinical settings. This result indicates excellent concordance between the CAMBRA and CAMBRA-OP questionnaires for risk assessment of caries.

The internal consistency of the two instruments used to assess caries risk, CAMBRA and CAMBRA-OP, was evaluated using Cronbach’s alpha. The results revealed an excellent internal consistency, with a Cronbach’s alpha of 0.972 (based on standardized items: 0.972) for the two-item scale. The interitem correlation was extremely high (r = 0.946), indicating a strong agreement between the two caries risk assessment methods. Descriptive statistics showed similar mean scores for both measures (CAMBRA = 2.38, SD = 1.06; CAMBRA-OP = 2.44, SD = 1.02).

The Chi-square test was performed, revealing a statistically significant association between the CAMBRA-OP Questionnaire caries risk and the qualitative variables related to children’s caries risk (Table 2).

A multinomial logistic regression was conducted, revealing a significant association between patients’ gender and salivary acidic pH, with a *p*-value of 0.039. The Plaque Index exhibited a notable relationship with salivary acidic pH, as did the patients’ dietary quality survey scores. Conversely, high sugar consumption and caries risk did not show clear associations with acidic salivary pH in this analysis. All additionally details concerning the statistical analysis are provided in the Supplementary Materials Section at the end of the main text. (Tables S1–S6).

## 4. Discussion

In this project, it was necessary to adapt the CAMBRA Questionnaire to clinical practice, eliminating the possibility of performing salivary bacterial cultures. This decision was made because such cultures were not feasible to implement or maintain in dental offices and university clinics due to financial and time constraints. The primary objective was to complete the questionnaires in the most realistic manner possible, considering the routine practice of pediatric dentistry.

The study sample comprised 300 healthy individuals aged between 6 and 14 years. The lower age limit was established for several methodological reasons. Specifically, the CAMBRA Questionnaire, used as the reference tool in this research, distinguishes only two age categories: 0 to 5 years and 6 years and older. The original CAMBRA protocol does not subdivide the population within the 6–14-year age range, which supports the decision to use a single questionnaire for all children in this age bracket. This approach ensured the homogeneity of the data collection instrument and guaranteed compatibility of the information obtained across the pediatric population.

There is considerable disparity in the literature regarding age ranges and sample sizes. Some studies cover very broad age ranges, from 3 to 17 years [27,28,29,30], whereas others focus on narrower groups, such as 6 to 12 years [21,29,31,32,33]. This variability complicates direct comparisons between studies but also highlights the need for tools that are adapted to specific age groups, particularly in pediatric dentistry.

### 4.1. Caries Risk Assessment Using the CAMBRA Questionnaire in the Pediatric Population

The CAMBRA Questionnaire is recognized as a valuable indicator of oral health and caries risk. It also emphasizes the critical role of primary caregivers, who are responsible for providing adequate education on dietary habits and oral hygiene, which are essential factors in preventing the onset and progression of dental caries. Therefore, caregivers constitute a central component in assessing caries risk and in implementing more effective preventive interventions.

Despite its utility, the application of the CAMBRA Questionnaire in the pediatric population presents significant challenges. One of these is that many questions are designed for adults and do not adapt to the characteristics and needs of the different age groups within childhood. This reduces the questionnaire’s ability in identifying clinical and behavioral particularities specific to children [34].

In adults, the CAMBRA Questionnaire has demonstrated consistent and reliable results across diverse populations and clinical contexts, allowing for the stratification of caries risk into low, moderate, or high categories [23,35,36,37,38,39,40,41,42,43,44]. For instance, Khattak et al. [37] evaluated individuals aged 14 to 30 years in Pakistan and Saudi Arabia, finding that 75.9% and 61.7% of participants, respectively, were classified as high risk. Similarly, Iqbal et al. [23] reported in 2022 that 85% of participants (aged 6–60 years) were at high risk of caries, while only 15% were classified as having moderate risk.

The CAMBRA Questionnaire has also been evaluated in educational and training contexts, demonstrating that it enables coherent stratification of caries risk and facilitates the guidance of preventive and non-surgical therapeutic interventions, such as the application of fluoride and chlorhexidine. Studies by Rechmann [35] Kaur [36], Chaffe [42] and confirmed its efficacy and reproducibility in adults of various ages, while Ramos-Gómez [40] demonstrated its usefulness in dental student training and clinical decision making. Pakdaman [39] also showed that the CAMBRA Questionnaire can be adapted to different healthcare systems and cultural contexts, consolidating it as a valid and stable tool for managing caries risk.

Taken together, these studies support the use of CAMBRA as a valid and adaptable instrument for assessing and managing caries risk in adults. Although risk levels vary between populations and regions, most authors agree that implementing a risk-based approach allows for more effective, rational, and efficient preventive interventions [21,26,33,34,35,36,37,38,42,43,44,45].

However, in children, the application of CAMBRA has notable limitations. Key determinants of oral health in childhood, such as habits acquired before the age of six, are not considered. Additionally, the questionnaire partially depends on the subjectivity of the professional, which can introduce bias. Its ability for detecting clinical and behavior particularities in children is low, and its adaptation to different sociocultural and socioeconomic contexts is limited, making its use outside the United States of America challenging. While valuable as a teaching tool, it may be overly theoretical for pediatric clinical practice. Consequently, any adaptation of CAMBRA for children should demonstrate high concordance and preliminary feasibility and should be integrated into a broader assessment that considers the child’s family environment [21,33].

### 4.2. Comparison with Previous Studies

Several studies have explored the application of the CAMBRA Questionnaire in the pediatric population. Iqbal et al. [23] and Aboubakr et al. [45] reported moderate (61.2%) and high (92.5%) caries risk, respectively. Although CAMBRA can be applied to children.

In our study, 60% of children examined had active caries, and 48.7% were classified as having high risk according to the CAMBRA Questionnaire. This reflects the frequent presence of active disease and associated risk factors, including habitual sugar consumption, poor oral hygiene, and family history of caries.

The adaptation of CAMBRA in the CAMBRA-OP Questionnaire addresses the main limitations of the original version by integrating behavior and familial factors and optimizing the assessment of protective and risk factors specific to childhood. This approach enhances clinical applicability, facilitates caregiver education, and enables the design of individualized preventive interventions.

Compared to Mateos et al. (2023) [33], whose questionnaire relies primarily on parental perception without direct clinical examination, the CAMBRA-OP Questionnaire combines clinical assessment with behavioral and familial information, thereby increasing ability. Similarly, Iqbal et al. [21,23], who applied CAMBRA to broad populations (6–60 years), did not specifically differentiate the pediatric population or incorporate familial and behavioral factors, limiting its relevance to pediatric dentistry.

Featherstone and Chaffee et al. in 2018 [17] emphasize CAMBRA’s usefulness for risk stratification and guiding preventive interventions but acknowledge that adaptations are required for children to improve clinical ability and relevance. The CAMBRA-OP Questionnaire meets this need, enabling application in clinics and early intervention centers through caregiver interviews and basic clinical examination.

While CAMBRA and other instruments, such as CANBRA [17,46,47], are effective for objective caries risk assessment, many do not systematically integrate behavioral and familial factors. Unlike previous studies, CAMBRA-OP allows for a more ability and realistic assessment of risk in children aged 6–14 years, prioritizing direct clinical information and omitting elements of limited relevance or difficult implementation.

### 4.3. Children’s Questionnaire Modified by the University of Seville (CAMBRA-OP Questionnaire)

The University of Seville developed a modified version of the CAMBRA-OP Questionnaire to address limitations in the original protocol for pediatric populations (Appendix A). The original CAMBRA was overly theoretical [9,16,17,20,21,46,47,48] and did not consider critical factors such as habits acquired before age six or the family environment.

Unlike the static original CAMBRA, it adopts a longitudinal perspective, allowing continuous monitoring of patient progress.

Validation showed a high statistical correlation between CAMBRA and the CAMBRA-OP Questionnaire (Cramer’s V = 0.826; *p* < 0.001) and excellent diagnostic agreement (Cohen’s Kappa = 0.815; *p* < 0.001). The extremely high Cronbach’s alpha value (α = 0.972) observed for the two-item scale indicates excellent internal consistency between the CAMBRA and CAMBRA-OP instruments. Furthermore, the very strong interitem correlation (r = 0.946) suggests a high degree of agreement between the two methods for assessing caries risk. These findings support the reliability of both instruments and indicate that they can be used interchangeably to evaluate caries risk in clinical and research settings. The results also reinforce the robustness of these tools, providing confidence in their capacity to consistently capture the same construct across different assessment formats. This finding indicates an increased ability of the CAMBRA-OP instrument in detecting pediatric patients (aged 6–14 years) who are at risk of caries. These results demonstrate consistent risk identification particularly for early risk profiles not captured by clinical signs alone.

A comparison of three widely used questionnaires, the original CAMBRA, the CAMBRA-OP Questionnaire, and Mateos et al. (2023) [33], revealed significant differences in approach. While CAMBRA focuses exclusively on clinical aspects, the CAMBRA-OP Questionnaire integrates behavioral and social factors, and Mateos et al.’s instrument relies [33] almost entirely on parental perception without direct clinical examination. CAMBRA-OP disease indicators are clinical and objective, including enamel lesions, dentine caries, and recent treatments, whereas parental reports in Mateos et al.’s study introduce subjectivity and potential bias.

Regarding risk factors, CAMBRA and CAMBRA-OP include items such as stimulated salivary flow and dental morphology. However, CAMBRA-OP further evaluates salivary pH, diet quality, and the modified Quigley-Hein plaque index, providing a more nuanced and comprehensive assessment of pediatric caries risk.

Children with systemic diseases, undergoing medical treatment, or receiving orthodontic care were excluded to ensure sample homogeneity, reducing bias. In contrast, previous studies included these cases without accounting for critical clinical elements, such as direct patient examination.

CAMBRA items of limited relevance for children, such as exposed roots, medication use, and bacterial cultures, were omitted in CAMBRA-OP, prioritizing salivary flow and pH measurements, in line with previous studies [46,47,48,49,50,51,52]. Questions regarding fluoridated water consumption were also removed due to negligible fluoride levels in participants’ residential areas.

Regarding protective factors, all three questionnaires emphasize brushing with fluoridated toothpaste. CAMBRA-OP, however, differentiates frequency and parental supervision and evaluates manual toothbrush use, brushing initiation before age six, and annual dental check-ups, which are factors not considered in other protocols. Additional fluoride products, such as arginine, calcium, or phosphate, were excluded, as parents were unfamiliar with them, highlighting the importance of adapting instruments to population knowledge and realities.

Overall, the CAMBRA-OP Questionnaire is statistically aligned with CAMBRA while providing a more clinically relevant, context-sensitive assessment. Moreover, the exclusion of low-relevance items and inclusion of more practical measures increases feasibility and acceptability in real world clinical settings. This refinement enhances practical implementation and reinforces its potential as a standardized tool for routine pediatric caries risk assessment (Table 3).

Disease indicators in CAMBRA-OP are clinical and objective, consistent with CAMBRA, including enamel lesions, dentine caries, and recent treatments. In contrast, Mateos et al.’s questionnaire [33] depends on parental memory and perception, introducing subjectivity and potential bias. Regarding risk factors, both CAMBRA and CAMBRA-OP include items such as stimulated salivary flow and dental morphology, whereas CAMBRA-OP further evaluates salivary pH, diet quality, and the modified Quigley-Hein plaque index, all strongly associated with caries risk (Table 3).

Children with systemic diseases, those undergoing medical treatment, or receiving orthodontic care were excluded to ensure sample homogeneity and reduce bias, as these conditions could alter key clinical parameters. By contrast, Mateos et al. [33] and CAMBRA included these cases but did not incorporate critical clinical elements, such as direct patient examination.

Instead, salivary flow and pH measurements were prioritized, consistent with previous studies facing similar challenges [50,51,52]. Additionally, CAMBRA-OP excluded questions regarding fluoridated water consumption, as participants resided in areas with negligible fluoride levels.

Regarding protective factors, all three questionnaires recognize the importance of brushing with fluoride toothpaste. However, CAMBRA-OP assesses this more precisely, differentiating frequency and parental supervision, while also including manual toothbrush use, brushing before age six, and the frequency of annual dental check-ups, factors not considered in other protocols. Additional fluoride products, such as arginine, calcium, or phosphate, were excluded, as parents were unfamiliar with these compounds, highlighting the necessity of adapting assessment tools to the knowledge and reality of the target population.

Despite demonstrating high concordance with CAMBRA and strong diagnostic consistency, the CAMBRA-OP Questionnaire’s effectiveness in reducing caries incidence and improving oral hygiene remains to be confirmed. Moreover, although it integrates behavior and familial factors, it relies partly on caregiver reports, which may introduce subjective bias. Certain protective factors, such as the use of additional fluoride compounds or xylitol intake, are not universally applicable or recognized by parents, which may reduce the efficacy of the questionnaire in specific populations.

## 5. Conclusions

The development of the CAMBRA-OP Questionnaire, specifically adapted to the pediatric population of Seville, has proven effective after obtaining solid evidence comparable to that of the original questionnaire. It represents a useful and objective tool that can be adapted to pediatric dentistry clinical practice. We determined caries risk in children aged 6 to 14 years, considering not only current clinical factors but also medical history, habits, and family environment. The results are reliable and statistically sound, allowing its conclusions to be extrapolated to the general population. This model not only improves risk assessment but also highlights the preventive work of pediatric dentists, highlighting the need for these types of questionnaires to be administered and supervised by properly trained professionals. The CAMBRA-OP Questionnaire underwent a validation process, confirming its validity and consistency.

## 6. Limitations and Future Perspectives

This study has several limitations that must be considered when interpreting the results. The lack of randomization in patient selection and the single-center design may have introduced selection bias and limited the generalizability of the findings. Moreover, the absence of a control group and the lack of longitudinal follow-up hinder the assessment of long-term outcomes and the sustained impact of interventions or parental knowledge on children’s oral health. Future studies should aim to include more diverse populations, including children with special needs, and protocols should be validated across multiple providers. Furthermore, the modified CAMBRA Questionnaire and its long-term predictive capacity warrant evaluation for adaptation in different regions. The development of a digital platform could facilitate the integration of data into clinical and academic practice, thereby promoting systematic prevention in pediatric dental care.

Nevertheless, longitudinal studies with control groups are required to evaluate its effectiveness in reducing caries incidence and improving oral hygiene habits. Such studies are essential to confirm that preventive interventions guided by the questionnaire produce measurable improvements in children’s oral health.

## Figures and Tables

**Table 1:** Frequencies and percentages of qualitative variables related to caries risk in children.

	Frequency (%)		
		Risk of Caries Based on CAMBRA Questionnaire	Risk of Caries Based on CAMBRA-OP Questionnaire
boys	Low	52 (32.1)	39 (24.2)
	Moderate	11 (6.8)	24 (14.9)
	High	81 (50.3)	81 (50.31)
	Extreme	17 (10.5)	17 (10.5)
girls	Low	47 (33.8)	44 (31.7)
	Moderate	10 (7.2)	13 (9.4)
	High	65 (47.8)	65 (47.8)
	Extreme	17 (12.2)	17 (12.2)
total	Low	99 (33)	83 (27.7)
	Moderate	21 (7)	37 (12.3)
	High	146 (48.7)	146 (48.7)
	Extreme	34 (11.3)	34 (11.3)

**Table 2:** Chi-square test showing the association between CAMBRA-OP Questionnaire caries risk and qualitative variables related to children’s caries risk.

CAMBRA-OP Questionnaire	*p*	Cramer’s V
CAMBRA Questionnaire	0.001	0.826
Modified Quigley-Hein plaque Index	0.001	
Diet quality survey	0.001	
Sugar consumption greater than 3 times/day	0.001	
Has the mother or primary caregiver had cavities in the past year?	0.002	
Has the child had any fillings in the past year?	0.001	
Has the child had dental visits in the past year?	0.001	
Does the child eat snacks or have sugary drinks between meals more than three times a day?	0.001	
Does the child regularly drink beverages other than water?	0.001	
Does the child brush their teeth with 1450 ppm fluoride toothpaste daily?	0.016	
Did the child brush their teeth three times a day before the age of 6?	0.028	
Do the parents check your child’s brushing at least once a day?	0.008	(*p* > 0.05)

**Table 3:** Comparison of different sections of the questionnaires used in the analysis.

	CAMBRA Questionnaire	CAMBRA-OP Questionnaire	Mateos et al. Questionnaire [33]
Disease Indicators	Caries lesions in dentin (Visual or radiographic diagnosis)	Caries lesions in dentin (Visual or radiographic diagnosis)	
	Caries lesions in enamel (Visual, Diagnodent, or radiographic diagnosis)	Caries lesions in enamel (Visual, Diagnodent, or radiographic diagnosis)	
	White spot lesions on smooth surfaces	Decalcifications. Enamel caries on smooth surfaces	
	Restorations performed in the last three years	Dental treatments performed in the last three years	
			Has your child been taken to the dentist for cavities and had a filling?
Risk Factors	Large amount of plaque on teeth	Plaque index (Modified Quigley-Hein Index)	
	Factors reducing salivary flow		Presence of saliva-reducing factors (medication, medical conditions)
	Inadequate stimulated salivary flow (<1 mL/min)	Hyposalivation (Deficient stimulated salivary flow < 1 mL/min)	
		Salivary pH	
	Deep occlusal pits and fissures	Presence of deep occlusal pits and fissures on teeth	
	Exposed roots		
	Consumption of more than three snacks between meals	Does your child have a poor or fair diet quality?	More than 3 snacks or sugary drinks between meals
	Orthodontic treatment fixed	Consumption of sugars between main meals more than 3 times/day	
		Use of bottle feeding or breastfeeding on demand at night	
		Child sleeps with a bottle or feeds on demand	Child sleeps with a bottle or feeds on demand
	Designer drugs		
			Child has developmental problems or special needs
	*Lactobacillus* and *Streptococcus* culture		
	Saliva buffering capacity		
			Parents/caregivers with limited knowledge of healthy oral habits
	CAMBRA Questionnaire	CAMBRA-OP Questionnaire	Mateos et al. Questionnaire [32]
Protective factors	Live in an area with fluoridated water		Child drinks fluoridated water
		Does the child attend annual dental checkups?	
		Use of manual toothbrush	
	Uses a daily fluoride rinse	Daily fluoride mouthwash 226 ppm once/day	
		Brushing with 1450 ppm fluoride toothpaste daily from age 6	
		Brushing with 1450 ppm fluoride toothpaste daily from age 6	
	Brushes with fluoride toothpaste at least once/day	Brushing with fluoride toothpaste once/day by the child	Child brushes with fluoride toothpaste at least once/day
	Brushes with fluoride toothpaste at least twice/day	Brushing with fluoride toothpaste three times/day by the child	Child brushes with fluoride toothpaste at least twice/day
		Parental supervision of tooth brushing from age 6	
		Parental supervision of tooth brushing before age 6	
			Child uses a calcium- and phosphate-containing toothpaste in the last six months
	Brushes daily with 5000 ppm fluoride toothpaste		
	Uses toothpaste with 1.5% Arginine		
	Semi-annual application of Chlorhexidine and Thymol varnish		
	Semi-annual application of fluoride varnish		Application of fluoride varnish in the last 6 months
	Has consumed 1 mg xylitol 5 times/day during the last 6 months		Mother/father/caregiver chews xylitol gum 2–4 times/day

## Data Availability

Original contributions presented in this study are included in the article and Supplementary Materials. Further inquiries can be directed to the corresponding author.

## Supplementary Materials

The following supporting information can be downloaded at: https://www.mdpi.com/article/10.3390/dj13110530/s1, Table S1: Caries Risk Distribution in Boys by Age Using CAMBRA and CAMBRA-OP Questionnaires. Table S2: Caries Risk Distribution in Girls by Age Using CAMBRA and CAMBRA-OP Questionnaires. Table S3: Overall Caries Risk Frequency by Questionnaire Type Across All Participants. Table S4: Multivariate Logistic Regression of Acidic Salivary pH and Caries Risk Factors. Table S5: Multinomial logistic regression between grouped salivary pH, acidic pH, and categorical variables related to caries risk. Table S6: Internal Consistency Comparison Between CAMBRA and CAMBRA-OP Questionnaires Using Cronbach’s Alpha.

## Appendix A

**Appendix A Children’s Questionnaire Modified by the University of Seville (CAMBRA-OP Questionnaire)**			
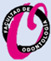
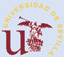
**Patient’s name:** **Age:** **Date:**			
**Disease Indicators**			
	**YES**	**YES**	**YES**
Dental caries in dentin (clinical or radiographic diagnosis)			
Dental caries in enamel (clinical or radiographic diagnosis)			
Demineralizations or enamel caries on smooth surfaces			
Dental treatments in the past 3 years			
**Risk Factors**			
Plaque index (Modified Quigley-Hein Index)			
Hyposalivation (low stimulated salivary flow—sialometry)			
Salivary pH			
Deep occlusal pits and fissures in teeth			
Poor or suboptimal diet quality			
Sugar intake > 3 times/day between main meals			
Bottle-feeding or on-demand breastfeeding at night			
Bottle-feeding or breastfeeding beyond 3 years of age			
Parent(s) had caries within the past year			
**Protective Factors**			
Daily toothbrushing with fluoride toothpaste (1450 ppm) from age 6			
Child brushes with fluoride toothpaste once/day			
Child brushes with fluoride toothpaste three times/day			
Parents supervise/assist brushing from age 6			
Daily use of fluoride mouthrinse (226 ppm) once/day			
Use of manual toothbrush			
Daily brushing with fluoride toothpaste (1000 ppm) before age 6			
Parents supervise/assist brushing before age 6			
Child attends annual dental check-ups			
**Total score:**			
**Final Result: Caries Risk: **			
