# Supplementary material for: Modified CAMBRA Protocol for Caries Risk Assessment in Children Aged 6 to 14 Years
Source: Dent J (Basel). 2025 Nov 12;13(11):530. doi: 10.3390/dj13110530 (PMC12651856; doi:10.3390/dj13110530)
Supplement: Supplementary file 1 [file dentistry-13-00530-s001.zip › dentistry-3909707-supplementary.pdf]

dentistry journal MDPI

Table S1	Caries Risk Distribution in Boys by Age Using CAMBRA and CAMBRA-OP Questionnaires		
	FREQUENCY (%) IN BOYS		
		Risk of caries by Questionnaire CAMBRA	Risk of caries by CAMBRA-OP Questionnaire
6 years	Low	8 (30,8%)	8 (30,8%)
	Moderate	3 (11,5%)	3 (11,5%)
	High	12 (46,2%)	12 (46,2%)
	Extreme	3 (11.5%)	3 (11.5%)
7 years	Low	*9 (33,3%)*	*5 (18,5%)*
	Moderate	*1 (3,7%)*	*5 (18,5%)*
	High	13 (48,1%)	13 (48,1%)
	Extreme	4 (14,8%)	4 (14,8%)
8 years	Low	6 (20,0%)	5 (16,7%)
	Moderate	1 (3,3%)	2 (6,7%)
	High	19 (63,3%)	19 (63,3%)
	Extreme	4 (13,3%)	4 (13,3%)
9 years	Low	5 (35,7%)	3 (21,4%)
	Moderate	1 (7,1%)	3 (21,4%)
	High	6 (42,9%)	6 (42,9%)
	Extreme	2 (14,3%)	2 (14,3%)
10 years	Low	4 (26,7%)	5 (33,3%)

	Moderate	4 (26,7%)	3 (20,0%)
	High	5 (33,3%)	5 (33,3%)
	Extreme	2 (13,3%)	2 (13,3%)
11 years	Low	7 (46,7%)	5 (33,3%)
	Moderate	0 (0,0%)	2 (13,3%)
	High	8 (53,3%)	8 (53,3%)
	Extreme	0 (0,0%)	0 (0,0%)
12 years	Low	3 (21,4%)	3 (21,4%)
	Moderate	1 (7,1%)	1 (7,1%)
	High	10 (71,4%)	10 (71,4%)
	Extreme	0 (0,0%)	0 (0,0%)
13 years	Low	6 (46,2%)	2 (15,4%)
	Moderate	0 (0,0%)	4 (30,8%)
	High	6 (46,2%)	6 (46,2%)
	Extreme	1 (7,7%)	1 (7,7%)
14 years	Low	4 (50,0%)	3 (37,5%)
	Moderate	0 (0,0%)	1 (12,5%)
	High	3 (37,5%)	3 (37,5%)
	Extreme	1 (12,5%)	1 (12,5%)
TOTAL BOYS	Low	*52 (32,1%)*	*39 (24,2%)*
	Moderate	*11 (6,8%)*	*24 (14,9%)*
	High	81 (50,3%)	81 (50,31%)
	Extreme	17 (10,5%)	17 (10,5%)

Table S2	Caries Risk Distribution in Girls by Age Using CAMBRA and CAMBRA-OP Questionnaires		
	FREQUENCY (%) IN GIRLS		
		Risk of caries by Questionnaire CAMBRA	Risk of caries by CAMBRA-OP Questionnaire
6 years	Low	6 (24,0%)	6 (24,0%)
	Moderate	1 (4,0%)	1 (4,0%)
	High	13 (52,0%)	13 (52,0%)
	Extreme	5 (20,0%)	5 (20,0%)
7 years	Low	1 (8,3%)	0 (0,0%)
	Moderate	1 (8,3%)	2 (16,7%)
	High	7 (58,3%)	7 (58,3%)
	Extreme	3 (25,0%)	3 (25,0%)
8 years	Low	4 (17,4%)	4 (17,4%)
	Moderate	0 (0,0%)	0 (0,0%)
	High	15 (65,2%)	15 (65,2%)
	Extreme	4 (17,4%)	4 (17,4%)
9 years	Low	8 (44,4%)	10 (55,6%)
	Moderate	4 (22,2%)	2 (11,1%)
	High	6 (33,3%)	6 (33,3%)
	Extreme	0 (0,0%)	0 (0,0%)
10 years	Low	8 (50,0%)	9 (56,3%)

	Moderate	2 (12,5%)	1 (6,3%)
	High	5 (31,3%)	5 (31,3%)
	Extreme	1 (6,3%)	1 (6,3%)
11 years	Low	8 (47,1%)	7 (41,2%)
	Moderate	1 (5,9%)	2 (11,8%)
	High	5 (29,4%)	5 (29,4%)
	Extreme	3 (17,6%)	3 (17,6%)
12 years	Low	6 (50,0%)	4 (33,3%)
	Moderate	0 (0,0%)	2 (16,7%)
	High	6 (50,0%)	6 (50,0%)
	Extreme	0 (0,0%)	0 (0,0%)
13 years	Low	4 (57,1%)	3 (42,9%)
	Moderate	1 (14,3%)	2 (28,6%)
	High	1 (14,3%)	1 (14,3%)
	Extreme	1 (14,3%)	1 (14,3%)
14 years	Low	2 (25,0%)	1 (12,5%)
	Moderate	0 (0,0%)	1 (12,5%)
	High	6 (75,0%)	6 (75,0%)
	Extreme	0 (0,0%)	0 (0,0%)
TOTAL GIRLS	Low	*47 (33,8%)*	*44 (31,7%)*
	Moderate	*10 (7,2%)*	*13 (9,4%)*
	High	65 (47,8%)	65 (47,8%)
	Extreme	17 (12,2%)	17 (12,2%)

Table S3 Overall Caries Risk Frequency by Questionnaire Type Across All Participants

		FREQUENCY (%)	
		Risk of caries by Questionnaire CAMBRA	Risk of caries by CAMBRA-OP Questionnaire
TOTAL	Low	99(33)	83(27,7)
	Moderate	21(7)	37(12,3)
	High	146(48,7)	146(48,7)
	Extreme	34(11,3)	34(11,3)

Table S4 Multivariate Logistic Regression of Acidic Salivary pH and Caries Risk Factors

pH SALIVAL. Acid pH		Wald	Sig.	OR	IC 95%
Gender	Boys	4,281	0,039*	0,560	0,324 – 0,970
	Girls	-	-	-	-
CAMBRA Questionnaire	Low	0,851	0,356	0,489	0,107 – 2,233
	Moderate	0,433	0,511	0,620	0,150 – 2,574
	High	0,058	0,809	0,891	0,351 – 2,264
	Extreme	-	-	-	-
CAMBRA-OP Questionnaire	Low	0,071	0,789	1,173	0,364 – 3,777
	Moderate	-	-	-	-
	High	-	-	-	-

	Extreme	-	-	-	-
Modified Quigley-Hein plaque Index	0	4,059	0,044*	0,082	0,082 – 0,966
	1	1,144	0,285	0,541	0,175 – 1,669
	2	1,058	0,304	0,558	0,184– 1,695
	3	-	-	-	-
Diet quality survey	Good	1300,179	<0,001*	2,065 E-9	7,890 E-9 – 5,402 E-8
	Needs Improvement	-	-	-	-
	Bad	-	-	-	-
Sugar consumption greater than 3 times/day	No	0,454	0,500	0,705	0,254 – 1,951
	Yes	-	-	-	-
			*(p<0,05)		

Table S5. Multinomial logistic regression between grouped salivary pH, acidic pH, and categorical variables related to caries risk

pH SALIVAL. Acid pH		Wald	Sig.	OR	IC 95%
Gender	Boys	4,281	0,039*	0,560	0,324 – 0,970
	Girls	-	-	-	-
CAMBRA-OP	Low	0,851	0,356	0,489	0,107 – 2,233
	Moderate	0,433	0,511	0,620	0,150 – 2,574
	High	0,058	0,809	0,891	0,351 – 2,264
	Extreme	-	-	-	-
CAMBRA	Low	0,071	0,789	1,173	0,364 – 3,777

	Moderate	-	-	-	-
	High	-	-	-	-
	Extreme	-	-	-	-
Modified Quigley-Hein plaque Index	0	4,059	0,044*	0,082	0,082 – 0,966
	1	1,144	0,285	0,541	0,175 – 1,669
	2	1,058	0,304	0,558	0,184– 1,695
	3	-	-	-	-
Diet quality survey	Good	1300,179	<0,001*	2,065 E-9	7,890 E-9 – 5,402 E-8
	Needs Improvement	-	-	-	-
	Bad	-	-	-	-
Sugar consumption greater than 3 times/day	No	0,454	0,500	0,705	0,254 – 1,951
	Yes	-	-	-	-
			**(p<0,05)*		

Table S6. Internal Consistency Comparison Between CAMBRA and CAMBRA-OP Questionnaires Using Cronbach's Alpha

	Mean Rank	SD	Cronbach's alpha	Interitem correlation
CAMBRA Questionnaire	2,38	1.06	0.972	0.946
CAMBRA-OP Questionnaire	2,44	1.02		
